# Multi-Omics Techniques Make it Possible to Analyze Sepsis-Associated Acute Kidney Injury Comprehensively

**DOI:** 10.3389/fimmu.2022.905601

**Published:** 2022-07-07

**Authors:** Jiao Qiao, Liyan Cui

**Affiliations:** ^1^ Department of Laboratory Medicine, Peking University Third Hospital, Beijing, China; ^2^ Core Unit of National Clinical Research Center for Laboratory Medicine, Peking University Third Hospital, Beijing, China; ^3^ Institute of Medical Technology, Peking University Health Science Center, Beijing, China

**Keywords:** sepsis-associated acute kidney injury, pathophysiology, biomarkers, omics database, multi-omics integration

## Abstract

Sepsis-associated acute kidney injury (SA-AKI) is a common complication in critically ill patients with high morbidity and mortality. SA-AKI varies considerably in disease presentation, progression, and response to treatment, highlighting the heterogeneity of the underlying biological mechanisms. In this review, we briefly describe the pathophysiology of SA-AKI, biomarkers, reference databases, and available omics techniques. Advances in omics technology allow for comprehensive analysis of SA-AKI, and the integration of multiple omics provides an opportunity to understand the information flow behind the disease. These approaches will drive a shift in current paradigms for the prevention, diagnosis, and staging and provide the renal community with significant advances in precision medicine in SA-AKI analysis.

## Introduction

The development of SA-AKI has been widely concerned but poorly understood in recent years, and its definition covers a heterogeneous group of diseases (1). In 2016, The Third International Consensus Definitions for Sepsis and Septic Shock (Sepsis-3) was proposed (2). Since then, SA-AKI has generally been defined as sepsis or septic shock involving the kidney, resulting in a progressive decline in renal function while meeting the Global Organization for Prognosis of Kidney Disease (KDIGO) CRITERIA for AKI and excluding other possible causes of renal impairment (3, 4). AKI and sepsis are defined using clinical symptoms (5). AKI is defined as loss of renal function, increased serum creatinine (SCr) levels, and/or decreased urine production (6); Sepsis is defined as a life-threatening organ dysfunction caused by uncontrolled infection and host reactions (7). Septic shock, a subset of sepsis, is strongly associated with a higher risk of death in circulatory, molecular, and metabolic abnormalities than sepsis alone. Patients with septic shock, characterized by hypotension, can be clinically identified by the need for antihypertensive agents to maintain mean artery≥65mmHg and serum lactic acid > 2mmol/L (>18mg/dL), excluding hypovolemia (8).

Currently, little is known about the epidemiology of SA-AKI. Adhikari et al. (9) extrapolated from incidence rates in the United States to estimate that there are as many as 19 million cases of sepsis per year worldwide. The annual incidence of SA-AKI may be about 6 million cases, or close to 1 case per 1,000 people, but the actual incidence is likely to be much higher. Although sepsis has long been recognized as the most common cause of AKI in critically ill patients, sepsis and its treatment may damage the kidneys. For example, a multinational, multi-center, prospective epidemiological survey showed that sepsis accounted for 45% - 70% of all AKI cases in intensive care units (10); However, AKI from any source was associated with a higher risk of sepsis, and Mehta et al. (11) found that 40% of severely ill patients developed sepsis after AKI, suggesting that AKI may increase the risk of sepsis. As individual syndromes, sepsis and AKI predispose hosts to each other, and it is often difficult to determine the exact timing of the onset of these two syndromes clinically.

Observational studies have shown that damage during SA-AKI occurs early in the critical course of illness and after admission to ICU. In a recent large cohort study, 68% of 5443 patients with septic shock developed evidence of AKI within 6 hours of the visit (12). However, AKI in late sepsis was associated with poorer clinical outcomes and increased mortality (76.5% compared with 61.5% in early AKI) (13). The high-risk group for SA-AKI is elderly patients, more common in women, and the combination of chronic kidney disease, diabetes, heart failure, malignant tumor, and liver disease will increase the susceptibility of patients to SA-AKI (14, 15). SA-AKI was strongly associated with adverse clinical outcomes compared with non-septic AKI. Firstly, Compared with non-septic AKI, SA-AKI was associated with higher severity scores, increased need for RRT, increased risk of death, and prolonged LOS (16). Secondly, SA-AKI was associated with a longer hospital stay (37 days vs. 21 days) and a gradual increase in hospital stay depending on the severity of AKI (17). Thirdly, long-term outcomes of SA-AKI patients depend on the severity of AKI and recovery status at discharge and have a similar prognosis to non-septic AKI (18). Finally, patients with recovered AKI are still at risk for chronic kidney disease (CKD), end-stage renal disease, and death, depending on the severity of AKI, RRT requirements, and recovery status during hospitalization. One study observed CKD at 1 year in 21%, 30%, and 79% of 105 survivors of AKI reversal, recovery, and non-recovery, respectively (19).

This review focused on integrating multiple types of omics data into SA-AKI studies. This review was divided into three parts. First, we outlined the pathophysiology and biomarkers of SA-AKI; Secondly, we discussed the application of multi-omics techniques in THE study of SA-AKI. Finally, we outline the new techniques and prospects of multi-omics approaches.

## Pathophysiological Mechanisms of SA-AKI

Our understanding of the pathogenesis of SA-AKI is limited. Much of the current understanding of SA-AKI has been extrapolated from animal models of sepsis, *in vitro* cell studies, and postmortem observations in humans with sepsis. Postmortem kidney biopsy samples from patients provide invaluable information about proper clinical conditions. The National Institutes of Health has launched programs such as the Kidney Precision Medicine Program to expand our understanding of AKI by obtaining kidney biopsies from patients with AKI to address AKI research’s technical and ethical limitations. SA-AKI animal models provide a wealth of observational data for complex and invasive measures not available in humans, such as monitoring renal blood flow (RBF), microvascular flow, cortical and medullary perfusion, oxygenation, and renal tubule health.

### SA-AKI Models

In mammals represented by mice and rats, there are three main SA-AKI modeling methods (20): (1) Direct endotoxin administration, in which lipopolysaccharide (LPS) is directly injected into the peritoneum or intravenously. LPS is a cell wall component of Gram-negative bacteria; (2) Cecum ligation puncture (CLP) or intraperitoneal implants of excrement and urine, similar model USES the ascending colon bracket, it allows the feces from the intestinal leakage to the peritoneum, CLP model induced sepsis is relatively easy, but with the severity of the sepsis, the amount and type of bacteria release is different also, does not necessarily lead to AKI. (3) The bacterial implant model is where bacterial impregnation is placed at the desired location (within the peritoneum or blood vessels), most commonly with fibrin clots. The most widely used animal models are the first two, in which inflammation occurs, microvascular permeability increases, and white blood cells are recruited; Hemodynamic parameters changed, GFR decreased, and renal function deteriorated. In all mammals (but most commonly used in large mammals (pigs and sheep) and zebrafish), direct bacterial delivery of live bacteria from Gram-negative and Gram-positive bacteria directly to the host (vein, peritoneal, subcutaneous, or directly into organs) is commonly used (20). In a recent prospective controlled study, the septic shock sheep model was widely used to study SA-AKI *in vivo* using Gram-negative bacteria and to assess renal function, histology, and glomerular ultrastructure in patients with septic shock (21). It overcomes the shortcomings of the endotoxin model and supports the view that early SA-AKI represents renal insufficiency.

The ideal animal model of sepsis should consistently translate relevant information from animal studies into the human condition. Rodents are small and relatively inexpensive, but the correlation between the mouse endotoxemia model and human gene change was very low and almost random (R ^2 =^ 0.01) (22). Compared with small animals, large animals such as pigs have similar cytokine and immune cell profiles and exhibit the characteristic symptoms of human infection (23). In addition, pigs are anatomically and physiologically similar to human kidneys and have obvious advantages in modeling operations. Pigs have a more macroscopic anatomical structure, the renal artery, renal vein and ureter can be easily separated during surgery, and instruments used in laparoscopic surgery for adults or children can also be used in miniature pigs. Therefore, pigs appear to be an appropriate animal model for SA-AKI.

A conference on What are the Microbial components involved in the pathogenesis of Sepsis held at Rockefeller University in May 1998 discussed the relative merits of the 2-hit hypothesis to explain the process of fatal septic shock and the “multi-hit” collaborative threshold hypothesis (24). The development of the 2-hit models allowed the researchers to determine the role of inflammatory mediators in susceptibility to post-injury infection and to create 2-hit models that replicate the clinical situation to generate different injury-specific inflammation patterns, from which to account for the complex interrelationships occurring in sepsis. 2-hit models of CLP and P. aeruginosa inoculation have been reported as clinically relevant sepsis models, J.m. Walker et al. (25)studied the possible beneficial effect of Specialized Pro-considerations Mediators (SPMs) given in the postsepsis stage to reduce infection/injury in a second blow. Results show that RvD2 Resolvin D2 (RvD2) promotes host defense by increasing TLR-2 signaling and macrophage/monocyte phagocytosis in less lethal and less inflammatory bacterial sepsis 48 h after the onset of sepsis. Jacqueline Unsinger et al. (26) examined IL-7, currently in several clinical trials (including hepatitis and human immunodeficiency virus), for improved survival in a 2-hit fungal sepsis model. Clinically relevant 2-hit models can provide a clearer understanding of the *in vivo* mechanisms of host defense in sepsis. While there are similarities in temporal inflammation and genomic host response patterns between humans and mice, the mouse immune system is more resistant. Human sepsis complications usually occur within a few days of trauma, and mice must be artificially created (27). In addition, it may vary depending on the type of injury and other variables such as outbreeding/inbreeding lines, rodent age, etc. Therefore, the similarity of the immune inflammatory blueprint in the 2-hit models between the animal and the patient should determine the timing of the impact (28).

The ideal animal model of sepsis should consistently translate relevant information from animal studies into the human condition. Currently, most animal studies use young, healthy models with no comorbidities. After searching for keywords (SA-AKI, models, comorbidities), there are very few literature studies on SA-AKI models associated with comorbidities such as advanced age, cardiovascular events, etc. One study used trauma/hemorrhage two-strike model (TH, first strike) and caecal ligation puncture model (CLP, second strike) in female (♀) and male (♂)CD-1 mice aged 3, 15, and 20 months. The study showed that age/sex differences in survival, while undeniably influential, were not reflected in the response patterns delineated between the corresponding groups. The exact role of gender/age in sepsis outcomes requires further experimental and clinical review. Another study was Kent Doi et al. (29)constructed 2-hit models of FA-CLP mice to replicate the clinical findings of high sepsis mortality in CKD patients. By introducing preexisting co-existence to mimic the common observation that human sepsis is more common in patients with underlying chronic diseases.

### Pathophysiological Mechanisms

**Figure 1:**
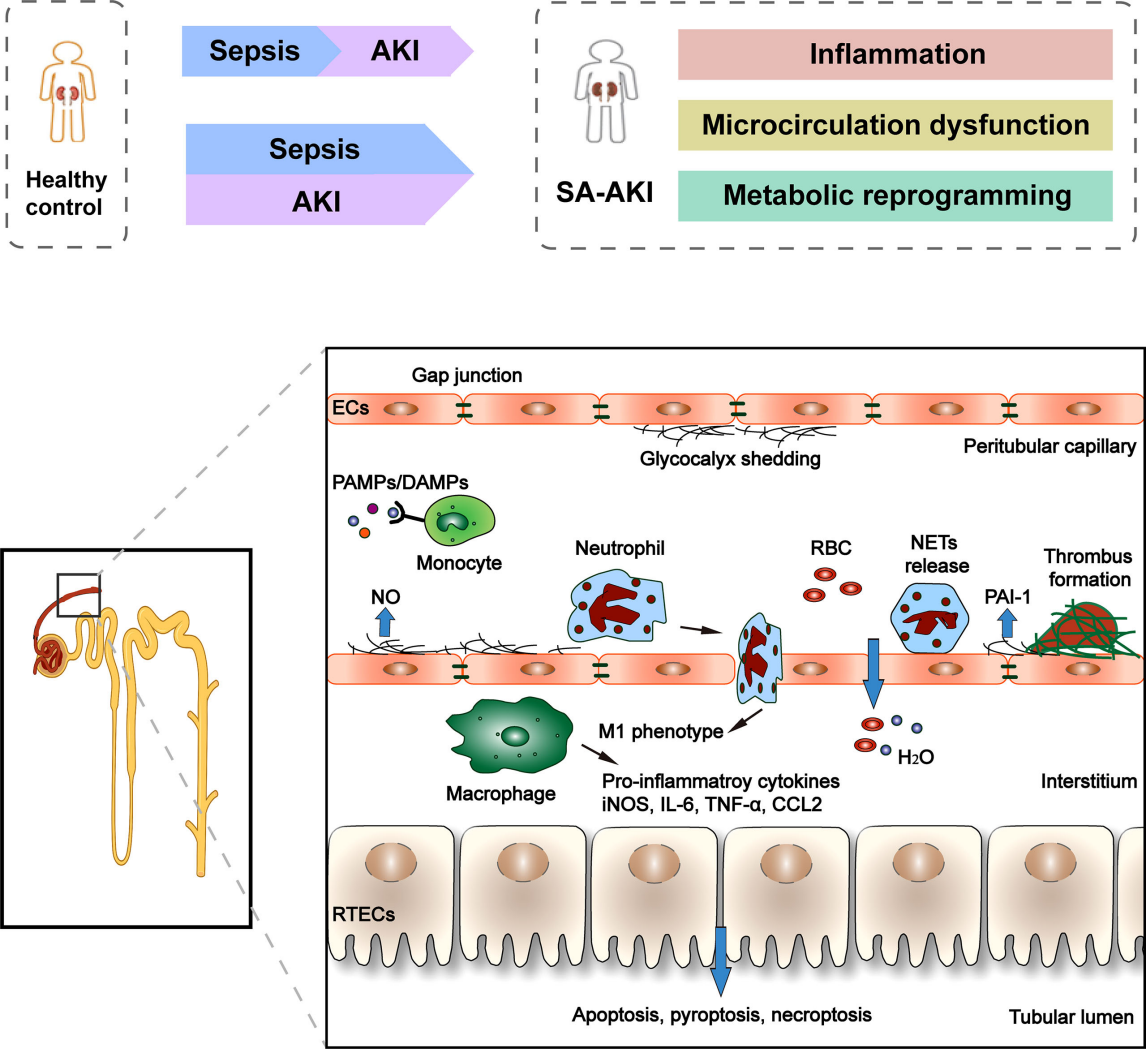
Clinical course and pathophysiology of SA-AKI. Sepsis is the most common cause of AKI in critically ill patients. However, sepsis and AKI predispose hosts to each other, and it is often difficult to determine the exact timing of the onset of these two syndromes clinically. There is increasing evidence that the pathogenesis of SA-AKI is the “unified theory” theory involving the interaction between inflammation, microcirculation dysfunction, and metabolic reprogramming. The pathophysiology of SA-AKI involves injury and dysfunction of many cell types, including macrophages, ECs, and RTECs. PAMP and/or DAMP released from damaged tissues activate and promote the pro-inflammatory phenotype (M1) of macrophages, resulting in the release of pro-inflammatory cytokines and chemokines, which can cause damage to kidney tissues. The second cell type that is vulnerable is the EC. Sepsis stimulates endothelial cells to produce nitric oxide, which causes blood vessels to dilate. Many molecules simultaneously control microvascular permeability, resulting in insufficient blood volume relative to the vessel when tight cellular connections loosen. In addition, during the period of sepsis, confirmed microvascular thrombosis related to inflammation. In RTECs, infiltration of inflammatory cells and a large number of inflammatory factors lead to deterioration of renal function, apoptotic cell death, and sublethal injury.

Since SA-AKI can occur in the absence of clinical symptoms of renal hypoperfusion and hemodynamic instability and the presence of normal or increased global renal blood flow, it is gradually recognized that ischemia-reperfusion injury is not the only mechanism of SA-AKI, and the “unified theory” theory is widely accepted (**Figure 1**). The pathophysiology of SA-AKI involves injury and dysfunction of many cell types, including macrophages, vascular endothelial cells (ECs), and renal tubular epithelial cells (TECs), as well as their crosstalk and association (30). There is increasing evidence that the pathogenesis of SA-AKI is multifactorial and complex, involving the interaction between inflammation, microcirculation dysfunction, and metabolic reprogramming.

#### Inflammatory and Immune Response

Dysregulated inflammation is the primary cause of many downstream complications, including kidney injury (31). In fact, the more significant the inflammatory response is more likely to lead to direct kidney damage. Macrophages play a central role in innate immunity (32). The first stage of the host response involves pathogen-associated molecular patterns (PAMP) binding to pattern recognition receptors (PRR) of innate immune cells, such as toll-like receptors, triggering downstream cascades of signals involved in early innate immune responses, leading to the synthesis and release of pro-inflammatory cell molecules and chemokines. Renal Tubular epithelial cells (RTECs) also express Toll-like receptors, especially TLR2 and TLR4 (33). A variety of cell-derived mediators release damage-related molecular patterns (DAMP) after tissue injury, promoting the pro-inflammatory phenotype (M1) of macrophages, activating the same sequence of events as PAMP amplifies the initial host response and affects local and distal cellular function, including proteolytic enzymes, reactive oxygen species (ROS), and neutrophil extracellular traps (NETs) (34, 35). During the progression of SA-AKI to CKD, resident cells with a specific phenotype undergo dedifferentiation, followed by proliferation and redifferentiation. Macrophages play an important role in this process. In addition to the proinflammatory phenotype described above, macrophages also have a profibrotic phenotype, stimulating fibroblasts and myofibroblasts, accompanied by the deposition of type I and III collagen and fibronectin. RTECs during repair may be involved in higher regenerative potential and anti-apoptotic ability (36).

#### Endothelial Injury and Microcirculation Dysfunction

The second cell type that is vulnerable is the EC. Sepsis stimulates endothelial cells to produce nitric oxide, leading to vascular dilation, loss of self-regulation, and endothelial dysfunction. Changes in cell-to-cell contact between endothelial cells are mediated by interactions between VEGF, VEGFR2, Ang, VE-cadherin, and ligand adhesion molecules, as well as complex interactions between endothelial cells and leukocytes that allow leukocytes to pass through (37). Many molecules simultaneously control microvascular permeability, resulting in insufficient blood volume relative to the vessel when tight cellular connections loosen. In addition, during the period of sepsis, confirmed microvascular thrombosis related to inflammation, bacterial pathogen associated molecular patterns were found in endothelial cells, platelets, and leukocytes on the surface of the PRR, bacterial endotoxin can also stimulate tissue factor expression and original activation increase fibrinolytic enzyme inhibitor 1 (PAI-1) levels, blocking fibrinolysis and subsequent initiation of the coagulation process promotes microvascular thrombosis (38, 39).

#### RETCs Apoptotic Cell Death and Sublethal Injury

In RTECs, infiltration of inflammatory cells and a large number of inflammatory factors lead to deterioration of renal function, apoptotic cell death, and sublethal injury. Sublethal changes in RTECs include loss of cell polarity, reduced tight junction protein expression, and biological energy disturbance (40). During the progression of SA-AKI to CKD, like immune cells, early metabolic reprogramming of TECs into aerobic glycolysis improves resistance and tolerance. In addition, epigenetic changes may occur, with cell cycle stagnation in the G2/M phase and a significant increase in connective tissue growth factor and TGF-β production (41).

#### Metabolic Reprogramming

Among the various cell types of the kidney, RTECs are the most metabolically active cells in the kidney and are very sensitive to septic-related injury. Under normal physiological conditions, oxidative phosphorylation (OXPHOS) produces more than 95% of the cellular energy of ATP (42), and aerobic respiration is the main mechanism of ATP production. However, during SA-AKI, RTECs may first convert to glycolysis, converting pyruvate to lactic acid, an inefficient mechanism for producing ATP. For example, CLP animal models and human SA-AKI lead to decreased ATP levels in the kidney (43). Inhibition of aerobic glycolysis and induction of OXPHOS can reduce susceptibility to AKI and significantly improve survival rate (44). As ATP levels decrease, adenosine monophosphate activated protein kinase (AMPK) activates, on the one hand leading to increased glycolysis, FA oxidation, and glucose transport capacity. On the other hand, it induces the production of key antioxidant enzymes and induces mitochondrial biogenesis through the peroxisome proliferator activated receptor (PPAR) γ CoActivator -1 α (PGC-1 α). Late activation of AMPK may eventually stabilize the energy balance through cell survival and mitochondrial biogenesis. The availability of functional mitochondria is an important component of cell metabolism and metabolic reprogramming. Sepsis results in significant mitochondrial damage and activation of mitochondrial quality control processes, such as mitochondrial autophagy (damaged mitochondria are swallowed into cells for recycling), biogenesis (new functional mitochondrial synthesis), or interference with cellular signaling pathways, such as the Akt/mTORC1/HIF-1 α pathway (45). Metabolic reprogramming may lead to optimization of RTECs energy consumption, reprogramming of substrate utilization, and enhanced cell resistance to oxidative damage (45). Therefore, the effect of OXPHOS induction or OXPHOS modulator promotion on mitochondrial function is closely related to renal function and survival rate during sepsis.

## The Diagnostic or Therapueitic Interventions of SA-AKI

The prevention of SA-AKI is complex, and most patients have already shown apparent renal insufficiency when seeking treatment. Therapeutically, SA-AKI remains largely supportive and nonspecific. Therefore, SA-AKI urgently needs to find more effective prevention and intervention methods. The past decade has seen an explosion in the use of high-throughput technologies and computational integration of multidimensional data. Integrating multi-omics studies offers a deeper understanding of the mechanisms of SA-AKI and the possibility of individualized treatment on an individual basis. Next, the existing prevention and treatment interventions for SA-AKI were discussed.

### Antibiotics and Source Control

Early and appropriate sepsis source control were associated with a reduced risk of AKI and a greater likelihood of renal recovery within 24 hours (15). Improved monitoring of host responses through the use of transcriptomic and/or metabolomic analysis describes several novel interventions targeting immunotherapy. An example of a promising but failed attempt is a drug targeting toll-like receptor (TLR). In addition, a new type of epigenetic therapy that regulates interference in the epigenetic process of gene transcription in immune cells during sepsis could be used to restore the possibility of immune function. Induction of immunity and reversal of immune paralysis by β -glucan, and direct pharmacological manipulation of epigenetic enzymes (46). SIRT1 inhibitor EX-527, a small molecule-SIRT1 binding site that shuts down NAD +, increases leukocyte accumulation in the peritoneum and improves peritoneal bacterial clearance, showing significant protective effects during abdominal sepsis in mice (47).

### Fluid Resuscitation

Fluid resuscitation is the cornerstone of septic shock management. An initial moderate infusion of resuscitation solution (30 mL/kg within the first 3 hours) was followed by dynamic measurements of fluid reactivity to determine the need for fluid or vasoactive agents. There is clear evidence that excessive resuscitation is also harmful in the case of AKI (48). However, complimentary analysis analysis of the ProCESS trial focused on renal outcomes up to 1 year and found that the use of early goal-directed therapy, alternative resuscitation, or conventional care did not affect the development of new AKI, AKI severity, fluid overload, RRT requirements, or renal function recovery (18).

### Vasoactive Agent

In the case of SA-AKI, several large multicenter trials have looked at traditional drugs such as norepinephrine (norepinephrine), epinephrine, vasopressin, and dopamine, as well as more novel drugs such as angiotensin II and levosimendan (49). Norepinephrine is recommended as the first-line agent for septic shock treatment, and vasopressin is the consensus first-line agent for septic shock treatment (50). A small subgroup analysis of patients treated with RRT showed that patients receiving angiotensin II required less RRT than placebo and were more likely to survive to day 28 (53% versus 30%; P=0.012), the results need to be validated in a larger SA-AKI cohort (51).

### Drug Therapy

Another treatment for sepsis is to protect individual organs. In preclinical and small clinical studies, recombinant human alkaline phosphatase (AP) has shown a protective effect against SA-AKI through direct dephosphorylation of endotoxin leading to reduced inflammation and organ dysfunction and improved survival (52). In a recent international, randomized, double-blind, placebo-controlled, dose-discovery adaptive Phase IIa/IIb study of 301 PATIENTS with SA-AKI, 1.6 mg/kg was found to be the optimal dose with no significant improvement in short-term renal function compared with placebo. However, the use of AP was associated with a reduction in day 28 mortality (17.4% versus 29.5% in the placebo group) (52).

Thiamine deficiency is associated with anaerobic metabolism and increased lactic acid. Adding thiamine improves mitochondrial function in sepsis. In a secondary analysis of a single-center, randomized, double-blind, placebo-controlled trial, patients randomized to intravenous thiamine (200 mg twice daily for 7 days) had lower AKI severity and fewer patients received RRT (53). Targeted therapies, such as targeting apoptotic pathways with caspase inhibitors and inhibiting inflammatory cascades, have shown some promising results in experimental models (54).

As of June 2022, a search at www.clinicaltrials.gov listed 2,772 sepsis studies, of which 94 SA-AKI studies and 49 involved intervention (clinical trials). Many other compounds are being actively investigated for sepsis, such as remtimod, pirfenidone sustained release, l-carnitine, and probiotics.

### Renal Replacement Therapy

Guidelines suggest using continuous or intermittent renal replacement therapy (weak recommendation, low-quality evidence) for patients with adult sepsis/septic shock who develop AKI and require RRT (2). Widely accepted indications for initiation of RRT include refractory fluid overload, severe hyperkalemia, and metabolic acidosis in which drug therapy fails, uremic signs (pericarditis and encephalopathy), dialyzable drug or toxicosis (55). There is little data on the effect of RRT initiation timing (early and delayed strategies) on SA-AKI. Early initiation of RRT may improve prognosis by limiting systemic inflammation, fluid overload, and organ damage, but there are currently no specific RCTs to determine the optimal time to initiate RRT in SA-AKI. In the RENAL and ATN studies, there was no significant difference in the odds ratio (OR) of mortality in patients with sepsis who received higher and lower intensities of RRT (56). In addition, SA-AKI has associated with lower SCr and more pronounced oliguria, so the less severe KDIGO stage defining these criteria may underestimate the severity of AKI and create a bias in the time to define RRT. New potential biomarkers that can predict AKI severity, such as TIMP-2 x IGFBP-7, may help determine when to start RRT in this setting (57).

## The Omics Era and its Impact on the Study of SA-AKI

SA-AKI is currently defined in terms of clinical symptoms, and there is considerable variation in disease presentation, progression, and response to treatment, highlighting the heterogeneity of the underlying biological mechanisms. As a result, clinicians encounter much uncertainty when considering the best treatment and risk prediction.

**Figure 2:**
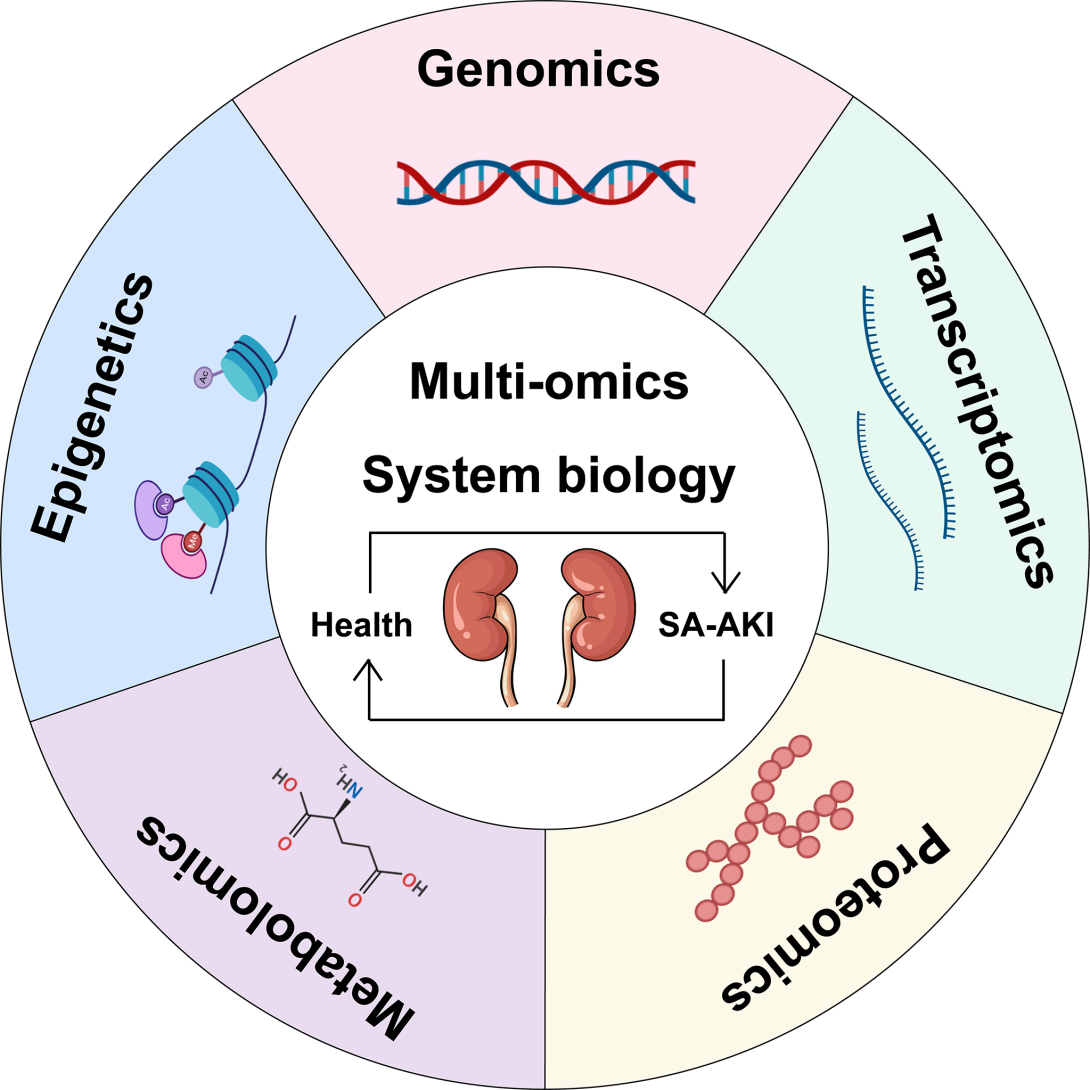
Schematic representation of a multi-omics approach to SA-AKI. Single omics data can be integrated into multiple-omics and combined with systems biology to understand the pathophysiological mechanisms of SA-AKI better and facilitate the discovery and development of emerging biomarkers for treatment.

Omics refers to the comprehensive study of the roles, relationships, and effects of various molecules in biological cells. Today, omics technologies are advancing rapidly, and large datasets can be obtained from individuals and patient populations of the SA-AKI genotype-phenotypic continuum (**Figure 2**). Starting with genomics, new sequencing technologies have been used rapidly elucidate entire genomes and simultaneously analyze all genes (58). There are also transcriptomics (the study of the expression of all genes in a cell or organism), proteomics (the analysis of all proteins), metabolomics (the comprehensive analysis of all metabolic small molecules), epigenomics, metagenomics, glycomics, lipidomics, connectomics, and so on (59). A fundamental shift in integrative biology from focusing on the function of individual molecules or pathways to analyzing biological systems as a unified whole is the direction in which omics technology is developing. Combined with these high-dimensional data sets, computational methods such as machine learning provide the opportunity to reclassify patients into molecularly defined subgroups that better reflect underlying disease mechanisms, with the ultimate goal of improving diagnostic classification, risk stratification, and allocation of molecular, disease-specific therapies for patients with SA-AKI. Therefore, we will first discuss the application of individual omics techniques to the study of SA-AKI (**Table 1**) and then provide a comprehensive use of multi-omics.

**Table 1:** Summary of omics studies of SA-AKI.

Study	Omic technology	Methods/models	Major findings
Frank, 2013 (60)	Genomics-DNA microarray	1,264 patients with septic shock, of these, 887 white patients were randomly assigned to the discovery and validation cohort	5 SNPs were associated with SA-AKI: BCL2, SERPINA, SIK3 genes.
Vilander, 2017 (61)	Genomics-SNP genotyping	2567 patients without chronic kidney disease and with a genetic sample, 837 had sepsis and 627 had septic shock	SERPINA4 and SERPINA5, but not BCL2 and SIK3 are associated with acute kidney injury in critically ill patients with septic shock.
Genga, 2018 (62)	Genomics-DNA polymorphisms	Two cohorts were retrospectively analyzed: Derivation Cohort (202 patients with sepsis enrolled at the Emergency Department from 2011 to 2014 in Vancouver, Canada); Validation Cohort (604 septic shock patients enrolled into the Vasopressin in Septic Shock Trial (VASST)).	CETP modulates HDL-C levels in sepsis. CETP genotype may identify patients at high-risk of sepsis-associated AKI.
Vilander, 2019 (63)	Genomics-Genotyping polymorphism	653 patients with sepsis, 300 had KDIGO stage 2 or 3 AKI and 353 did not (KDIGO Stage 0)	Association between short repeats of HMOX1 and AKI risk in sepsis patients.
Sun, 2020 (64)	Genomics-SNP genotyping	235 patients with AKI and 235 patients without AKI (No AKI)	SNPS in NFKB1 loci rs41275743 and RS4648143 are associated with the risk of AKI in sepsis patients.
Tran, 2011 (65)	Transcriptomics-gene expression microarray	Mice after intraperitoneal injection of LPS (Global knockouts and mice with the floxed PGC-1α allele have been previously described)	Restored expression of the mitochondrial biogenesis factor PGC-1α appears to be necessary for recovery from endotoxemic AKI.
Basu, 2011 (66)	Transcriptomics-gene expression microarray	179 children with septic shock and 53 age-matched normal controls	There were 21 unique gene probes upregulated in patients with SSAKI compared to patients without SSAKI.
Ge, 2017 (67)	Transcriptomics-gene expression microarray	Patients with sepsis induced AKI (n = 6), patients without sepsis AKI (n = 6), and healthy volunteers (n = 3)	miR-4321; miR-4270 were significantly upregulated in the sepsis-induced AKI compared with sepsis-non AKI, while only miR-4321 significantly overexpressed in the sepsis groups compared with control groups.
Hultström, 2018 (68)	Transcriptomics-gene expression microarray	Six high-quality microarray studies of renal gene expression after AKI	5,254 differentially expressed genes in at least one of the AKI models; MYC may be a central regulator of renal gene expression in tissue injury during AKI.
Tod, 2020 (69)	Transcriptomics-gene expression microarray	Mice after intraperitoneal injection of LPS	MiR-762 expression was significantly increased in the early stages of septic AKI, and clusters of miR-144/451 were upregulated at 24 h.
Holly, 2006 (70)	Urine proteomics-DIGE, MS	Rat CLP sepsis	A potential biomarker and drug target, Meprin-1-alpha, was identified in a septicaemic induced ARF rat model.
Maddens, 2012 (71)	Urine, plasma, tissue proteomics gel-free technique	Mice uterus ligation and E. coli inoculation	Urinary chitinase 3-like protein 1 and -3 and acidic mammalian chitinase discriminated sepsis from sepsis-induced AKI in mice.
Wu, 2015 (72)	Tissue proteomics-DIGE, MALDI-TOF/TOF MS	Mouse CLP sepsis	Phosphorylated MYL12B can be used as a potential plasma biomarker for early diagnosis of SA-AKI.
Hinkelbein, 2017 (73)	Tissue proteomics-DIGE, MS	Rat CLP sepsis	MUP5 decreased in SA-AKI. Mitochondrial energy production and electron transport were found to be significantly correlated with proteins.
Hashida, 2017 (74)	Proteomics from hemofilter adsorbates- SDS PAGE, MS	20 patients with AKI on ICU admission and who received continuous renal replacement therapy (CRRT) as usual care between June 2012 and March 2014 were studied.	Three proteins, including carbonic anhydrase 1 (CA1) and leucine-rich α -2-glycoprotein (LRG1), were identified in all samples from patients with sepsis compared with those without sepsis.
Li, 2020 (75)	Urinary proteomics-MS	Rat CLP sepsis; Sepsis was validated in human patients	PARK7 and CDH16 have been identified as novel biomarkers for the early diagnosis of septic AKI.
Lin, 2020 (76)	Tissue global Proteomic and Phosphoproteomic-SDS PAGE, MS	Mouse CLP moderately severe sepsis	2,119 protein and 2950 phosphorus sites were identified; Several new and/or less studied S-AKI labeled proteins Hmgcs2 Serpin S100a8 and Chil3 were validated.
Waltz, 2016 (77)	Tissue (whole kidney) metabolomics-LC/MS	Mice CLP sepsis	CLP induced renal injury as evidenced by elevated serum creatinine, blood urea nitrogen, and cystatin C. Global energetic profile in sepsis showed an increase in glycolytic intermediates with decreased flux through the tricarboxylic acid (TCA) cycle. Multiple inflammatory markers were elevated in response to CLP. Levels of osmotic regulators varied, with an overall increase in pinitol, urea, and taurine in response to CLP.
Li, 2017 (78)	Tissue and serum metabolomics-1H NMR	Mice after intraperitoneal injection of LPS	Obvious decreases in betaine, taurine, lactate, glucose, and significant increases in 3-CP, acetoacetate, pyruvate, NADPH, creatine, creatinine, TMAO in LPS mice.
Rodrigues, 2018 (79)	Urine metabolomics-NMR	Rat CLP sepsis	1H nuclear magnetic resonance analysis detected important increases in urinary creatine, allantoin, and dimethylglycine levels in septic rats. However, dimethylamine and methylsulfonylmethane metabolites were more frequently detected in septic animals treated with 6G or 10G, and were associated with increased survival of septic animals.
Garcia, 2019 (80)	Tissue, plasma, and urine metabolomics-NMR	Pigs infused with E. coli	Metabolic differences between control animals and septicemic animals: In renal tissue, lactic acid and niacin increased, while valine, aspartate, glucose and threonine decreased; Iso-glutamate n-acetyl glutamine n-acetyl aspartic acid and ascorbic acid increased, while inositol and phenylacetyl glycine decreased in urine; And In serum, lactate alanine pyruvate and glutamine increased, while valine glucose and betaine concentrations decreased.
Ping, 2019 (81)	Tissue metabolomics-GC-TOFMS	Rat after intraperitoneal injection of LPS	Metabolic disorders of taurine, pantothenic acid, and phenylalanine and phenylalanine in the renal cortex are associated with the development of SA-AKI.
Lin, 2020 (82)	Plasma metabolomics-GC/MS	Plasma samples from 31 patients with sepsis and 23 healthy individuals.	The downregulated energy, amino acid, and lipid metabolism found in our study may serve as a novel clinical marker for the dysregulated internal environment, particularly involving energy metabolism, which results in sepsis.

### Introduction of Omics Techniques and the Application in SA-AKI

#### Genomics

Genomics is used to identify individual genetic variation and disease susceptibility and studies relatively few individual heritable traits at specific loci. The completion of the Human Genome Project led to the initial sequencing of > 20,000-30,000 genes in the human genome, while current genomic studies have whole-genome sequencing, including regulatory regions and other untranslated regions, to identify potentially pathogenic variations anywhere in the genetic code; Whole exome sequencing, involving the sequencing of protein-coding regions of the genome, is a widely used next-generation sequencing (NGS) method. The human exome makes up no more than 2% of the genome, but it contains about 85% of the variants known to be associated with disease, making this approach a cost-effective alternative to whole genome sequencing; DNA microarrays rely on nucleic acid hybridization to detect the presence of SNPs and CNVs (83).

Studies have used large-scale genomic approaches to identify SNPs using microarrays of known variations in specific diseases to identify genetic variants associated with SA-AKI. Angela J. Frank et al. (60) included 1264 patients with septic shock, of whom 887 white patients were randomly assigned to the discovery and validation cohort, and found that 5 SNPs were associated with SA-AKI, such as BCL2, SERPINA, and SIK3 genes. Subsequently, Vilander (61) and colleagues included genetic samples from 2567 patients without chronic kidney disease, including 837 cases of sepsis and 627 cases of septic shock, and found that SERPINA4 and SERPINA5, but not BCL2 and SIK3 are associated with acute kidney injury in critically ill patients with septic shock. In addition to focusing on variants that influence survival after SA-AKI, the second goal is to find variants that influence SA-AKI risk. Laura M. Vilander et al. (64) found that SNPs in NFKB1 loci rs41275743 and RS4648143 are associated with the risk of AKI in sepsis patients.

#### Epigenomics

Epigenomics regulates gene transcription through epigenetic changes such as DNA methylation, histone modification, and changes in non-coding RNA expression. Binnie (84) and colleagues performed epigenome-wide DNA methylation analysis on whole blood samples from 68 sepsis and 66 non-sepsis severely ill adults and found 668 differential methylation regions (DMR), of which the majority (61%) were hypermethylated. SA-AKI research is currently focused on animal studies. Selective IIa class HDAC inhibitor TMP195 may have renal protective effects in LPS-induced SA-AKI mouse models (85). In LPS-induced AKI, down-regulation of miR-29B-3p exacerbates podocyte damage by targeting HDAC4. miR-29b-3p may be an important target for AKI therapy (86). Future research into the mechanisms of sepsis may aim to integrate epigenomics and transcriptome to determine how much variation in transcriptome is influenced by methylation, histone modifications, and non-coding transcripts.

#### Transcriptomics

Transcriptomics is the study of complete gene transcripts or RNA types that are transcribed by specific cells, tissues, or individuals at specific times and states (87). It includes both coding RNAs that are translated into proteins and non-coding RNAs that are involved in post-transcriptional control, which further affect gene expression. Unlike genomics, which focuses on static DNA sequences, transcriptomics can identify genes and gene networks that are activated or suppressed under specific conditions to assess dynamic gene expression patterns. At the quantitative level, with reference, genes could be quantitatively analyzed, while without reference, only Unigene (optimized transcript) could be quantitatively analyzed, and downstream differential gene analysis and functional annotation could be performed. At the structural level, parameters can be used for variable clipping, SNP analysis, gene structure optimization, and new gene prediction. At present, it has been widely used in basic research, clinical diagnosis, drug development, and other fields.

Mei Tran et al. (65) performed microarray sequencing after intraperitoneal injection of LPS in mice and found that restoring the expression of mitochondrial biogenic factor PGC-1α was necessary for the recovery of endotoxin AKI, indicating that changes in gene expression pathways related to cell metabolism and mitochondrial function were most abundant in septic LPS mice. In a study of 179 children with septic shock and 53 age-matched normal controls, Rajit K Basu et al. (66) found that 21 unique gene probes were upregulated in SA-AKI patients compared with non-SA-AKI patients. In other microarray experiments using miRNAs (non-translational RNA molecules with transcriptional regulatory functions), Qin-min Ge et al. (67) found that miR-4321 and miR-4270 were significantly upregulated in septic induced AKI compared with non-septic AKI, while only miR-4321 was significantly overexpressed in the septic group compared with the control group. Pal Tod et al. (69) observed that miR-762 expression was significantly increased in early septic AKI and the miR-144/451 cluster was upregulated at 24 h after intraperitoneal injection of LPS in mice.

#### Proteomics

With the development of omics technology, research has shifted to the analysis of translation “products” of cellular proteins and RNA transcripts. By mRNA transcription and protein modification after translation (add or remove phosphate or methyl specific molecular etc.) make the proteome is highly dynamic, can not necessarily infer from the level of gene expression in specific protein level, greatly increased the complexity of protein and peptide, has brought the huge challenge for proteomics analysis (88). The introduction of a variety of techniques, such as two-dimensional gel electrophoresis, liquid chromatography, and mass spectrometry, with high sensitivity and resolution mass spectrometry, has enabled the identification and quantification of proteins and peptides in tissues and biological fluids and has provided new insights into disease-related processes at the molecular level (89).

High-throughput proteomic analysis of urine, plasma, and tissue samples has identified emerging biomarkers and drug targets. To construct a new rat model of acute renal failure induced by sepsis with heterogenous response similar to that in humans. DIGE was used to detect changes in urinary protein and identify potential biomarkers and drug targets for Meprin-1-alpha (70); MUP5 decreased in SA-AKI, and mitochondrial energy production and electron transport were significantly correlated with protein (73). PARK7 and CDH16 are considered novel biomarkers for early diagnosis of septic AKI and validated in human patients (75). In the Mouse CLP Sepsis model, the Tissue Proteomics-Dige and MALDI-Tof/TOF MS techniques were used to identify the phosphorylated MYL12B as a potential plasma biomarker for the early diagnosis of SA-AKI (72). Tomoaki Hashida et al. studied 20 AKI patients hospitalized in ICU from June 2012 to March 2014 by Proteomics from Hemofilter Bates SDS PAGE, MS. Three proteins, including carbonic anhydrase 1 (CA1) and leucine-rich α-2-glycoprotein (LRG1), were detected in samples from all sepsis patients who received continuous renal replacement therapy (CRRT) under conventional care, compared with those who were not infected with sepsis (74).

Several recent studies have identified several promising candidate marker proteins for disease onset and progression, and further identified pathways specific to SA-AKI and its transition to CKD. The moderate and severe mouse CLP sepsis models were established, and the time changes of kidney proteomics and phosphorylated proteomics were examined on days 2 and 7 after surgery, and 2119 protein sites and 2950 phosphorus sites were identified. Several new and/or less studied SA-AKI labeled proteins Hmgcs2, Serpin S100A8, and Chil3 were validated (76). In the migration and E. coli inoculation model, Using proteomics Gel-free technique, urine chitinase 3-like proteins 1, 3 and acidic mammalian chitinase were found to distinguish between sepsis and mouse septicaemic induced AKI, NGAL, and thioredoxins, and increased with the severity of AKI (71).

#### Metabonomics

Metabolomics refers to the comprehensive and systematic identification and quantitative analysis of molecular metabolites of less than 1000 daltons in biological samples such as blood and tissues under physiological or pathological conditions, which may more accurately describe the cellular processes active under any conditions (90). Metabolomics studies use two main methods to detect metabolites: nuclear magnetic resonance (NMR) and liquid chromatography/mass spectrometry (LC/MS). NMR is quantitative, non-destructive, reproducible, and can accurately quantify the abundance and molecular structure of metabolites (91). The sample preparation is simple and the measurement time is relatively short, which is suitable for high-throughput, untargeted metabolite fingerprint study. But the disadvantage is relatively low sensitivity. LC/MS is also widely used in metabolomics, with higher sensitivity and quantification of more metabolites, but with poor accuracy and reproducibility. Sample preparation and solvent selection are even more critical in MS-based experiments because metabolite extraction requires the removal of proteins and salts that adversely affect the quality of the measurement as well as the instrument itself. MS mass analyzers in metabolomics commonly use quadrupole time of flight, Orbitrap, and Fourier transform, which are suitable for distinguishing the chemical complexity of metabolomics (92).

Metabolites are the final products of biological activities and are the most direct and comprehensive biomarkers reflecting physiological phenotypes. More and more studies have shown that changes in energy metabolic pathways, also known as metabolic reprogramming, are an important factor in the pathophysiology of SA-AKI. Therefore, it is of great significance to study the metabolic changes of SI-AKI and identify its early biomarkers for early clinical diagnosis and treatment. Firstly, inflammatory metabolites and products of kidney damage increase. Paul Waltz et al. (77) used metabolomics-LC/MS technology in the MICE CLP Sepsis model and found that the evidence of CLP-induced kidney injury is increased serum creatinine, blood urea nitrogen, and cystatin C. CLP raises multiple inflammatory markers. Levels of osmotic regulators varied, with an overall increase in pinitol, urea, and taurine in response to CLP. Francisco Adelvane de Paulo Rodrigues (79) and colleagues detected significant increases in creatine, allantoin, and dimethylglycine levels in septic rats by 1-hour NMR analysis. However, dimethylamine and methanosulfonyl metabolites were detected more frequently in septic animals treated with 6-gingerol (6G) and 10-gingerol (10G) and were associated with increased survival in septic animals. Gingerol alleviates septic AKI by reducing renal dysfunction, oxidative stress, and inflammatory response, and the mechanism may be related to the increased production of dimethylamine and methanosulfonyl methane.

Secondly, the overall energy spectrum of sepsis showed an increase in glycolysis intermediates and a decrease in flux through the tricarboxylic acid (TCA) cycle. Similar changes in metabolites were also observed through tissue and serum metabolomics-1H NMR after LPS injection in mice. The contents of betaine, taurine, lactic acid, and glucose in LPS mice were significantly decreased. The contents of 3-CP, acetoacetic acid, pyruvate, NADPH, creatine, creatinine, and trimethylamine oxide were significantly increased (78). In large animal models of pigs infused with E. coli, metabolic differences were found between control and sepsis animals: lactic acid and niacin increased in renal tissues, while valine, aspartic acid, glucose, and threonine decreased; The contents of isoglutamate-acetylglutamate-acetylaspartic acid and ascorbic acid in the urine increased, while the contents of inositol and phenylacetyl glycine decreased. Serum concentrations of lactic acid, alanine, pyruvate, and glutamine increased, while those of valine, glucose and betaine decreased (80).

In addition, in plasma samples from 31 patients with sepsis and 23 healthy individuals, metabolomics-GC/MS suggest that down-regulation of energy, amino acid, and lipid metabolism may serve as a new clinical marker for identifying internal environmental disorders, especially involving energy metabolism, leading to sepsis (82).

### Omics Techniques in SA-AKI Outcome Events

Through literature review, SA-AKI is important to the two topics cited above and is considered a major public health problem associated with increased mortality and progression to CKD. However, relevant studies are mainly focused on prospective and observational cohort studies of patients in the real world, and there are still few studies on omics technology. Omics, especially multi-omics, may have more in-depth exploration and analysis of the two topics, which is the direction of further research of omics technology in SA-AKI.

While maintaining homeostasis, the kidney, as an endocrine and immune organ, may regulate distant multi-organ dysfunction. Several recent experimental studies have shown that AKI is associated with extensive damage to distant organs such as the lungs, heart, liver, and intestines (93, 94). The function of remote organs can be affected by a variety of biologically related pathways, such as transcriptome changes, apoptosis, upregulation of various damage promoting molecules, oxidative stress, inflammation, and loss of vascular function (95). In addition, the severity of organ dysfunction is independently associated with mortality, which can rise to as high as 45%-60% when AKI is associated with other organ dysfunction, such as acute respiratory distress syndrome [ARDS], heart failure, or sepsis. In a prospective observational cohort of 1753 patients with critically ill AKI, SA-AKI (n = 833) was associated with an increased risk of in-hospital death. In a systematic review of long-term renal outcomes after septic AKI and long-term renal outcomes, studies using keywords associated with septic AKI were identified from PubMed and CINAHL databases within 5 years, with a time range of 28 days to 3 years for long-term renal outcomes, Most take one year. Renal outcomes range from recovery to renal replacement therapy to death. All of these studies excluded patients with CKD (96).

The molecular mechanisms underlying AKI’s transformation into CKD are complex, and most literature has focused on the complex balance between adaptation and maladaptive repair processes (97). Maladaptive repair leads to chronic damage and loss of kidney function, setting the stage for CKD, which eventually progresses to ESRD. This process is accompanied by permanent changes in undesirable structures, persistent low-grade inflammation, activation of perivascular and interstitial fibroblasts, vascular sparseness, and parenchymal ischemia (98). The integration of multiple omics techniques opens up new possibilities for improving our understanding of AKI and the driving forces behind the transition from AKI to CKD. Yi-han Lin et al. (76) analyzed the changes in the global proteome and phosphorylated proteome levels in renal tissues on day 2 and day 7 after CLP by constructing a mouse model of moderate severity CLP and using filter-based sample processing method combined with an unlabeled quantitative method, corresponding to SA-AKI and transition to CKD, respectively. It provides a view that renal tissue dynamically regulates the oxidative stress induced by sepsis, and provides enlightenment for the exploration of potential diagnosis and treatment methods in the future. In this study, a total of 2119 proteins and 2950 phosphates were identified to identify specific response pathways to SA-Aki-CKD transformation, including regulation of cellular metabolism, oxidative stress, and energy expenditure in the affected kidney. Of these, the majority (56%) are associated with small molecular metabolic processes (FDR = 3.35E-48), such as lipids, nucleotides, alcohol, and other fatty acids. Network analysis also revealed that several protein clusters, such as REDOX enzyme complex, peroxisome, and cytochrome P450 (CYP) family proteins, may play important roles in the AKI-CKD transition.

### Novel Biomarkers for SA-AKI

The role of emerging biomarkers in different renal syndromes, including SA-AKI, is a rapidly growing area of research. In patients with sepsis, early detection of AKI is critical to provide optimal treatment and avoid further kidney damage. Because specific biomarkers can detect renal stress or damage before significant changes in function (preclinical AKI) or even before the absence of functional changes (subclinical AKI), studying SA-AKI biomarkers could provide additional insights into the pathophysiology of SA-AKI (99). In order to provide prevention and early diagnosis of treatment when it is most effective. **Table 2** summarizes some of the biomarkers studied in SA-AKI from the aspects of inflammatory, endothelial injury, tubule injury, and AKI risk markers, to provide prevention and early diagnosis when treatment is most effective.

**Table 2:** Summary of biomarkers used to detect SA-AKI.

Types of biomarker	Biomarker	Source	Potential use in SA-AKI
Inflammation biomarkers	IL-6	Mononuclear macrophages, Th2 cells, vascular endothelial cells and fibroblasts	Baseline IL-6 at admission predicted AKI in patients with severe sepsis, and IL-6 also predicts the development of AKI and need for RRT in patients with severe sepsis (100).
	IL-18	Monocytes, dendritic cells, macrophages and epithelial cells	In a prospective, multicenter cohort, UIL-18 independently predicted the progression of septic AKI (AUC 0.619; 95% CI, 0.525 to 0.731) (101).
	sTREM-1	Activated receptors selectively expressed on the surfaces of neutrophils, macrophages, and mature monocytes	In patients with sepsis, The AUC values of plasma STREM-1 in the diagnosis and prediction of AKI (24h before diagnosis) were 0.794 and 0.746, respectively. The AUC values of urine STREM-1 were 0.707 and 0.778. ACU 0.922 was predicted 48 hours before diagnosis, and urine STREM-1 was a fairly good predictor (102).
Endothelial injury biomarkers	Ang	Ang1 is mainly synthesized by paravascular sertoli cells, vascular smooth muscle cells and tumor cells; Ang2 is mainly synthesized by vascular smooth muscle cells	Ang1 has a protective effect against endotoxemia, increasing vasoconstriction and reducing pulmonary microvascular leakage associated with inflammation (103). Circulating Ang1 levels were suppressed in critically ill patients with septic shock (104). Circulating Ang-2 is a strong independent predictor of mortality in ICU dialysis-dependent AKI patients (105).
	VE-cadherin	Vascular endothelial cell	Plasma sVE-cadherin was independently associated with AKI-RRT, suggesting that disruption of endothelial adhesion and connectivity may contribute to the pathogenesis of organ dysfunction in sepsis (106).
	sTM	Vascular endothelial cell	Compared with sepsis non-AKI group, sTM in SA-AKI group was significantly different (P<0.0001); Multivariate logistic regression analysis showed that sTM was an independent predictor of AKI, and AUROC was 0.758(P<0.0001) (107).
Tubular injury biomarkers	NGAL	Leukocytes, loops of medullary and collecting ducts	SA-AKI patients have higher detectable plasma and urine NGAL compared with non-septic AKI patients. These differences in NGAL values in SA-AKI may have diagnostic and clinical relevance as well as pathogenetic implications (108).
	KIM-1	RTECs	UKIM-1 and sKIM-1 levels were significantly higher in SA-AKI than in patients without AKI. ROC of uKIM-1 and sKIM-1 for AKI prediction was 0.607 and 0.754, respectively (109).
	L-FABP	Liver cells; RTECs	Urinary L-FABP level may be a predictive marker of sepsis severity and mortality, and can serve as a useful biomarker for patients with sepsis complicated with AKI (109).
	Cys C	All nucleated cells	Urine and plasma are of value in the diagnosis and prediction of AKI occurrence (24 hours before diagnosis) in patients with SA-AKI (21). Aydogdu et al. confirmed that plasma and urine Cys-C were good markers for early diagnosis of septic associated AKI (AUCs 0.82 and 0.86, respectively) (110). However, some studies in adults and newborns have shown that sepsis has no effect on plasma or urine levels of Cys-C (111, 112).
AKI risk biomarkers	[TIMP-2] • [IGFBP7]	TIMP-2 is synthesized by RTECs; IGFBP7 is expressed in almost all epithelial cells	[TIMP-2] • [IGFBP7] predicted the development of stage 2 and 3 AKI in high-risk and critically ill patients with sepsis with an AUC of 0.84. The biomarker performed similarly regardless of disease severity (SOFA score), with a sensitivity of 77.5% and specificity of 75% for severe AKI at a cut-off value of 1.0 (113).
	Electronic alerts, electronic risk algorithms	\	Several alarms have shown the ability to predict sepsis and AKI separately, and the combination of biochemical biomarkers may help enrich the detection and risk stratification of SA-AKI (20).

IL-6, Interleukin-6; IL-18, Interleukin-18; sTREM-1, Soluble triggering receptor expressed myeloidcells 1; Ang, Angiopoietins; sTM, Soluble thrombomodulin; NGAL, Neutrophil gelatinase-associated lipocalin; KIM-1, Kidney Injury Molecule-1; L-FABP, Liver-type fatty acid binding protein; Cys C, Cystatin C; TIMP-2, Tissue Inhibitors Of Metalloproteinase 2; IGFBP7, Insulin-like growth factor binding protein-7.

Renal tubular cell damage contributes to the spread of AKI during sepsis. Among the newer biomarkers, neutrophil gelatinase-associated lipid carrier protein (NGAL), kidney injury molecule-1 (KIM-1), liver-type fatty acid binding protein (L-FABP), and cystatin C(Cys C) accelerated the diagnosis of SA-AKI. NGAL is the most widely studied renal biomarker which is a member of the human lipid carrier protein family and consists of 178 amino acid residues (114). The level of NGAL increased sharply after kidney injury, which can be used as an early sensitive biomarker of kidney injury. NGAL expression is inconsistent in SA-AKI. Studies have shown that urinary NGAL has higher specificity for S-AKI than plasma NGAL (80.0% vs 57.0%) (115). Sollip Kim et al. found in A systematic review and meta-analysis that plasma NGAL had A high sensitivity and A high negative predictive value for AKI in adult sepsis patients. However, this study did not reveal the usefulness of urine NGAL (116). KIM-1 is a type I transmembrane glycoprotein encoded by the TIM-1 gene and is a member of the T cell immunoglobulin mucin (TIM) gene family. Kim-1 was first used as a biomarker for acute kidney injury (AKI) in 2002, but there is little evidence to support its role in S-AKI. Similar to uKIM-1, sKIM-1 can also predict the occurrence of septic AKI at an early stage, but it has no predictive value to judge the severity of AKI and the prognosis of sepsis (109). However, these biomarkers lack the ability to further the stratification of SA-AKI risk or inform us of primary and secondary sites of injury.

Tissue Inhibitors Of metalloproteinase 2 (TIMP-2) stimulate P27 expression (117). Insulin-like growth factor binding protein-7 (IGFBP7) increases the expression of p53 and P21 (118). [TIMP-2]•[IGFBP7] (a biomarker of cell cycle arrest) accurately predict stage 2-3 AKI as defined by KDIGO within 12 hours; Induced stagnation of the renal tubule cell G_1_ cycle occurs simultaneously with early cell damage caused by ischemia or inflammatory processes. Changes in urine [TIMP-2]•[IGFBP7] after initial fluid resuscitation identified different risks of AKI progression in sepsis patients in the ProCESS trial of 688 septic shock patients at [TIMP-2]•[IGFBP7] before and after 6-hour resuscitation. According to the APACHE II score, clinical response to resuscitation was weak in predicting endpoint (AUC 0.68, 95%CI 0.62-0.73), It was improved by the addition of [TIMP-2]•[IGFBP7] (0.72, 95%CI 0.66-0.77 P =0.03) (119).

There are no widely accepted risk scores for SA-AKI, and only the HELENICC score currently predicts mortality in patients requiring renal replacement therapy (RRT) (120). Comparing 30 patients before electronic alert activation with 30 patients after electronic alert activation, the time to receive any sepsis-related intervention was shorter after an alert, with a median difference of 3.5 hours (P = 0.02) (121). Using electronic health records to create electronic alert systems has the potential to identify high-risk patients and initiate interventions more quickly. Using real-time data from electronic health records to identify patients with SA-AKI, automatic alarms are combined with biochemical biomarker testing to improve case detection and risk stratification for SA-AKI (122).

### Omics Databases on Kidney Disease

Omics database provides the latest information about the molecular function orientation and expression, to store information about has conducted a similar experiment, is helpful to study design, the study of kidney disease is a valuable tool. For clinical practice, systems biology methods and high throughput technology to promote medical revolution from passive to active and prevention, through the powerful calculation method, find new biomarkers. The development of diagnostic tools to elucidate the pathogenesis and create models for possible therapies for patient screening, diagnosis, prevention and treatment. Omics is ongoing and is expected to be gradually introduced into clinical practice within the next decade (123). In this review, referring to Theofilos Papadopoulos et al. (124), we describe universal omics databases covering a wide range of molecular and pathological information as well as specific databases for kidney disease (**Table 3**).

**Table 3:** General omics and Kidney-specific databases.

Tool	Data types	Purpose
**Genomics**		
GeneCards (http://www.genecards.org/)	Contains >152 000 GeneCards genes; Gene-centric data from approximately 150 network sources; Detailed information on all annotated and predicted human genes	Search for human genetic information; The first key to the study of gene function
Online Mendelian Inheritance in Man (http://www.omim.org/)	>15 000 genes; Contains a central database of information and literature on human genetic diseases and genetic loci	Looks for the latest clinical testing standards and trends;Provides detailed information about this class of annotations from gene sequences, maps, literature and other databases
NephQTL (http://www.nephqtl.org)*	Compartment-specific (glomeruli and tubulointerstitium) gene expression profiles from biopsy samples of 187 participants in the NEPTUNE cohort; Genotype frequency of SNPs in the NEPTUNE cohort	Compartment-specific (glomerular and tubule) eQTL discovery
Human Kidney eQTL Atlas (http://www.susztaklab.com/eqtl)*	Compartment-specific (glomeruli and tubulointerstitium) gene expression profiles from biopsy samples of 151 participants linked to SNP data	Compartment-specific (glomerular and tubule), as well as whole kidney eQTL discovery
**Transcriptomics**		
Gene Expression Omnibus (GEO) (http://www.ncbi.nlm.nih.gov/gds)	3848 data sets based on arrays and sequences; Storage of chips, second-generation sequencing and other high-throughput sequencing data	Gene expression data repository, data analysis and visualization
ArrayExpress (https://www.ebi.ac.uk/arrayexpress/)	60,054 high-throughput experiments; Storage of chips and high-throughput sequencing data	Arrayexpress database for EBI, similar to GEO database; Archives of Functional Genomics
Expression Atlas (https://www.ebi.ac.uk/gxa/)	1572 data sets; Provides information on gene expression patterns under different biological conditions	More focus on baseline trials
miRBase (http://www.mirbase.org)	28 645 miRNA entries from 223 species; Details of all published and annotated miRNAs	Databases used to study miRNA
Nephroseq (https://www.nephroseq.org/)*	26 data sets (1989 samples); Transcriptome analysis of biopsy samples from patients with renal disease; Clinical metadata from patients	Identifying disease-related signatures; Correlation of gene expression with clinical features
Renal Gene Expression Database(RGED) (http://rged.wall-eva.net/)*	88 research papers analysed; Contains comprehensive gene expression data sets from kidney disease studies	Contains comprehensive gene expression data sets from kidney disease studies; Provides a user-friendly utility for the nephropathy research community
Rebuilding a Kidney Consortium (http://www.rebuildingakidney.org)*	scRNA-seq visualizations from kidney biopsy samples, model systems and organoids; Shared resources with GUDMAP Lab protocols; Antibody validation; Cataloguing of iPSC lines	Coordinate studies and data relevant to nephron regeneration; Primary data access
Nephrocell (http://nephrocell.miktmc.org/)*	scRNA-seq data from kidney biopsy samples and organoids	Cell-selective gene marker identification
**Proteomics**		
PRoteomics IDEntifications (PRIDE) (https://www.ebi.ac.uk/pride/archive/)	Store 3342 items for protein/peptide identification, post-translational modifications, and supporting spectral evidence; One of the most prominent data repositories for proteomic data based on mass spectrometry (MS); Allows viewing of 2d gels and query scores	To enable the proteomics community to share publicly or in private partnerships the vast amounts of data generated by proteomics laboratories around the world.
Human Protein Atlas (http://www.proteinatlas.org/)	213 tissue and cell line samples; Proteomic analysis based on 24,028 antibodies against 16,975 unique proteins	To understand the expression of human protein in tissues, cell localization and pathological expression.
Human Kidney and Urine Proteome Project (HKUPP) (http://www.hkupp.org/)*	Look for proteins in renal structures (glomerulus, human medulla) and urine; Allows viewing of 2d gels and query scores	To promote proteomics research in the field of nephrology to better understand kidney function and the pathogenesis of kidney disease, and to define new biomarkers and therapeutic targets.
Urinary Protein Biomarker Database (http://122.70.220.102/biomarker)*	> 400 reports on humans and animals; 819 human biomarkers and 33 animal biomarkers were collected from the published literature	To obtain urinary protein specific biomarker candidates
**Metabolomics**		
Human Metabolome Database(HMDB) (http://www.hmdb.ca/)	Contains experimental MS/MS data for more than 5,700 compounds, experimental 1 H and 13 C NMR data (and allocation) for more than 1,300 compounds, and GC/MS spectral and retention index data for more than 780 compounds;	Obtain detailed information about metabolites and their associations with pathways, proteins, and reactions
Kyoto Encyclopedia of Genes and Genomes (KEGG)	One of the most complete and widely used databases containing metabolic pathways (495 reference pathways) from a variety of organisms (>4,700)	Understand a repository of advanced features and utilities for biological systems;
(https://www.kegg.jp)	>17,000 compounds, 10,000 drugs and nearly 11,000 glycan structures	With powerful graphics function, more intuitive and comprehensive understanding of metabolic pathways
Metabolite Link (Metlin) (https://metlin.scripps.edu)	>240,000 metabolites and 72,000 high resolution MS/MS spectrograms; A repository of mass spectrometry metabolite data	Emphasis should be placed on the identification of non-targeted Metabolomics metabolites in liquid
The Small Molecule Pathway Database(SMPDB) (http://smpdb.ca/)	910 hand-painted small molecule metabolic pathways, including 468 drug pathways, 232 disease pathways, 105 metabolic pathways and more than 100 other pathways; A database of interactive, visual small molecular pathways	Cleverly detailed hyperlinked diagrams of human metabolic pathways, metabolic disease pathways, metabolite signaling pathways, and drug activity pathways
**Multi-omics**		
Multi-Omics Profiling Expression Database (MOPED) (http://moped.proteinspire.org)	Absolute and relative protein expression data from more than 250 large-scale experiments; >500,000 proteomic absolute and relative expression records; >500,000 proteomic absolute and relative expression records; Relative gene expression data	Used to query different types of omics expression data and data visualization; View expression data, pathway mapping, and direct connections between proteins and genes; Provides a background for the exploration of multiple omics expressive data
Kidney Systems Biology Project (https://hpcwebapps.cit.nih.gov/ESBL/Database/)*	Transcriptomic, protein, Chip-seq data from model systems and renal epithelial cells	Gene- and protein-centred queries in kidney tissues, cells and segments
Kidney and Urinary Pathway Knowledge Base (KUPKB) (http://www.kupkb.org/)*	> 220 experiments; Contains data from renal and urinary high-throughput experiments, with rich links to other biological data, forming a single integrated repository	Collect open omics data related to kidney disease

## Multi-Omics Integration

Numerous studies have shown that the integration of multi-omics data sets has been applied to a wide range of biological problems, helping to unravel the underlying mechanisms at the multi-omics level. Yehudit Hasin et al. (125) proposed a comprehensive analysis method of multiple sets of data, which is divided into three categories: genomic priority that attempts to determine the mechanism of GWAS loci leading to disease, the phenotypic priority that seeks to understand the pathway leading to disease, and the environmental priority that uses environment as the primary variable to study its interfering pathway or interaction with genetic variation. Although current omics research on SA-AKI has focused on a single omics study, only a few studies have integrated multiple omics techniques to address the three critical issues of SA-AKI.

① Subtypes and classification of SA-AKI based on multiple omics features.

There has not been a multi-omics integrated study involved.

② Prognostic biomarkers for SA-AKI, including disease diagnosis and driver genes.

A good example is Raymond J. Langley et al. (126) and his colleagues examined clinical characteristics and plasma metabolomics and proteomics of patients with community-acquired sepsis upon arrival at the hospital emergency department and 24 h later. Different characteristics of proteins and metabolomics are concentrated in fatty acid transport and β -oxidation, gluconeogenesis, and citric acid cycles and vary more as death approaches. However, the metabolomics and proteomics of survivors of mild sepsis were not different from those of survivors of severe sepsis or septic shock. An algorithm derived from clinical features and measurements of seven metabolites predicted patient survival.

③Gain insight into the pathophysiology of SA-AKI.

Takashi Hato and his colleagues conducted two experiments specifically targeting SA-AKI. A combination of transcriptomics, proteomics, and metabolomics, showed that endotoxin preconditioning reprogrammed macrophages and tubules to create a protective environment to prevent severe AKI in septic mouse models, upregulating the antibacterial molecule itaconic acid and its activase Irg1. Many genes activated by endotoxin were located near heterochromatin, suggesting that epigenetic regulation may be involved in the preconditioning response (127). In the second study, they used gram-negative sepsis model for the translation group, transcriptome, and proteome of the joint inspection new; translation will be closed as a vital characteristic of the late sepsis, further found that 5 ‘cap dependency translation close the reversal of the improved degree of kidney damage caused by sepsis.

Mariam P. Alexander et al. (128) compared COVID-19 AKI with SA-AKI, and analyzed the morphological, transcriptome, and proteomic characteristics of postmortem kidneys. Transcriptomics found that COVID-19 AKI and SA-AKI have a rich transcriptional pathway associated with inflammation (apoptosis, autophagy, major histocompatibility complex I and II, and Type 1 T-assisted cell differentiation) compared to non-infectious AKI; Proteomic pathway analysis showed that both of them were enriched to a lesser extent in necrotic apoptosis and Sirtuin signaling pathways, both of which are involved in the regulatory response of inflammation.

## New Techniques and Future Perspectives

Our understanding of disease processes will likely to evolve rapidly and revolutionarily as new technologies and methods development. For example, techniques such as scRNA-seq and mononuclear RNA-seq (snRNA-seq) provide insights into the molecular processes of SA-AKI at the cellular level, with artificial intelligence aimed at accurately predicting the onset of SA-AKI in advance. In future applications, tissue samples or whole organs can be sequentially analyzed through a combination of these techniques to generate spatial multi-omics datasets, which are expected to provide unprecedented insights into the deep molecular biology of the system under study.

### Integrating Microarray-Based Spatial Transcriptomics and Single-Cell RNA-Seq

ScRNA-seq provides detailed information on single-cell transcriptional expression, allowing cell-to-cell analysis of RNA expression differences (129). It uses a variety of methods for cell isolation and transcription amplification, such as microfluidics devices that capture cells in hydrogel droplets or methods that rely on physical isolation of a cell (such as fluorescent-activated cell sorting into a 96-well plate and microfluidics chip used by Fluidigm C1) from another well (130). Due to the heterogeneous cell types (such as epithelial cells, endothelial cells, fibroblasts, vascular smooth muscle, and immune cells) in different renal microenvironments and interactions, SA-AKI has different effects on various cells in the kidney. scRNA-seq enables researchers to detect highly variable genes (HVGS) between cells that contribute to mixed populations, which cannot be achieved by bulk RNA-seq (131). One of the significant challenges of the scRNA-seq data is matching the RNA profile with its location (spatial information) in the tissue (132). Spatial transcriptome sequencing provides complete tissue spatial location information, enabling spatial localization of different single-cell subpopulations by adding spatial information to scRNA-seq data, increasing understanding of specific cell subpopulations and their interactions in development, homeostasis, and disease (133).

Currently, there are few studies on single-cell RNA sequencing technology for SA-AKI. Ricardo Melo Ferreira et al. (134) used single-cell sequencing to deconvolution the signature of each spatial transcriptome point in the mouse CLP model to determine the co-localization mode between immune cells and epithelial cells. Spatial transcriptomics revealed that infiltrating macrophages dominate the exocortical features, and Mdk was identified as the corresponding chemokine, revealing the mechanisms driving immune cell infiltration and detecting associated cell subsets to complement single-cell sequencing. Danielle Janosevic et al. (135) provided a detailed and accurate view of the evolution of renal endotoxemia at the cellular and molecular levels by sequencing single-cell RENAL RNA in a mouse endotoxemia model, providing the first description of spatio-temporal endotoxin-induced transcriptome changes in the kidney. It reveals that the involvement of various cell populations is organized and highly coordinated in time, promoting the further investigation of human sepsis.

### Artificial Intelligence

Artificial intelligence (AI) technology has emerged as doctors face the challenge of being overwhelmed by the amount of data generated in healthcare today (136). Artificial intelligence is a scientific discipline that aims to understand and design computer systems that display intellectual processes (137). Machine learning (ML), a subset of artificial intelligence, may detect disease onset before clinical symptoms appear, allowing for a more proactive approach (138). In machine learning, supervised learning and reinforcement learning are widely used (139).

In the narrative review of the clinical application of artificial intelligence in sepsis, 15 articles about the use of AI model to diagnose sepsis, the model with the best performance reached 0.97 AUROC; 7 prognostic articles, predicting mortality over time with an AUROC of up to 0.895; 3 articles on helping to treat sepsis, in which AI use was associated with the lowest mortality (140). Kumardeep Chaudhary et al. (141) used deep learning to identify the septicemic AKI subtype unknowingly and inexplicably from routinely collected data from electronic health records is the first study to use routinely collected electronic health record data to identify the clinical subtype of SA-AKI syndrome in the ICU. When combined with other biomarkers and omics data, this approach could further accelerate research into the discovering of new biomarkers and dysregulation pathways for SA-AKI.

At present, the comprehensive performance evaluation of machine learning models is limited by research heterogeneity. In addition, because clinical implementation of models is rare, there is an urgent need to determine the clinical impact on different patient populations to ensure universality (142).

## Conclusions

Despite significant advances in our understanding of the pathophysiology and detection markers of SA-AKI, it remains a common and highly hazardous complication of the critically ill disease. The development of multiple omics studies, which have increased the availability of kidney tissue, blood and urine samples, and patient data, has provided a tremendous opportunity to increase our understanding of SA-AKI. As the cost of omics analysis continues to decrease, the emergence of more types of omics techniques and studies integrating multiple omics techniques can be integrated into the clinic and guide the personalized treatment of SA-AKI. Such advances, however, will require a more careful selection of models and research techniques to study the effects of this molecular involvement on SA-AKI in greater detail, addressing the common challenges of omics in distinguishing causal and reactive changes in the context of disease.

## Author Contributions

JQ designed and wrote the review, drew the figures and tables. LC revised this manuscript and reviewed the figures and tables. All authors contributed to the article and approved the submitted version.

## Funding

National Natural Science Foundation of China (61771022), Key Clinical Specialty Funding Project of Beijing.

## Conflict of Interest

The authors declare that the research was conducted in the absence of any commercial or financial relationships that could be construed as a potential conflict of interest.

## Publisher’s Note

All claims expressed in this article are solely those of the authors and do not necessarily represent those of their affiliated organizations, or those of the publisher, the editors and the reviewers. Any product that may be evaluated in this article, or claim that may be made by its manufacturer, is not guaranteed or endorsed by the publisher.
